# High Q-factor reconfigurable microresonators induced in side-coupled optical fibres

**DOI:** 10.1038/s41377-023-01318-9

**Published:** 2023-11-23

**Authors:** Radan Slavík

**Affiliations:** Optoelectronics Research Centre, University of Southampton, Southampton, SO17 1BJ UK

**Keywords:** Optics and photonics, Applied optics

## Abstract

Recently, significant efforts have been devoted to enable light resonating inside various resonators for long time, leading to high Q factors. Achieving tunability of the free spectral range while maintaining high Q has been, however, challenging.

Interferometers are in the core of the most sensitive experiments carried out in science, ranging from famous Michelson–Morley interferometer that proved inexistence of aether to the recent proof of existence of gravitational waves. As interferometer’s resolution generally scales with the accumulated light phase (commonly characterized by the Q-factor, which is usually roughly proportional to the accumulated light delay), multi-path interferometers based on resonators such as Fabry–Perot or ring resonator are very popular, providing large delays in a small form factor. Resonators are also often behind a key tool that emerged from optical resonators: Optical Frequency Combs (OFCs).

When it comes to the question of how an ideal resonator would look like, one of the key items on the “wish list” is its tunability, in particular its spectral period, a.k.a. free spectral range (FSR) or comb spacing in OFCs. Tunability would allow to tayilor the resonator for various fields or enable its continuous change, of interest in a range of applications such as optomechanics^1,2^ or lasing^3,4^. However, FSR tunability requires the resonator’s length to be altered, e.g., when targeting one order of magnitude tunability, the length should be changeable by an order of magnitude. This is a requirement that most resonators, especially those with very high Q, struggle with.

In the newly published paper in *Light: Science & Applications*, Victor Vassiliev with Misha Sumetsky from the Aston Institute of Photonic Technologies, Aston University, Birmingham, UK, have proposed how to make FSR tuneable in a particular configuration of high-Q coupled microresonators^5^. To enable this, they chose very flexible platform in which two 3D microresonators are formed in two optical fibres, each exciting so-called Surface Nanoscale Axial Photonics (SNAP) microresonator^6^, Fig. 1. These are a special type of bottle resonators, where the bottle is formed by sub-micron fibre radius changes along the length due to the fabrication imperfections. The authors use two degrees of freedom to achieve wide continuous tuneability in FSR (over an order of magnitude) while keeping high Q factor (in excess of 1 million), which is very challenging to achieve for any high-Q resonators. These two degrees of freedom are the bend radius, which modifies the SNAP resonator shape and relative distance between the two fibres.

**Fig. 1:**
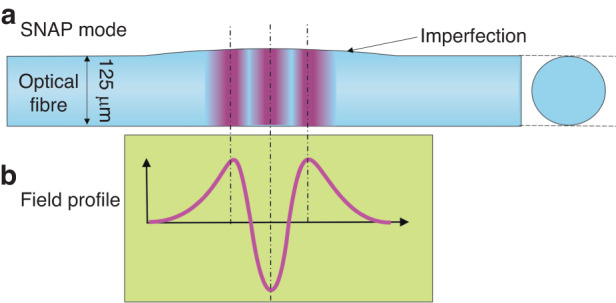
Schematic of a SNAP resonance. SNAP is a bottle-type resonator made by sub-micrometre fibre diameter imperfection (**a**). Here, resonator’s resonance with axial number of 3 is shown (**a**) together with its field profile (**b**). Compared to a simple whispering gallery mode resonance (axial number of 1), the shown mode extends into the 3rd dimension (fibre axial dimension), making it sensitive to fibre bending. Exciting two of such resonances in two closely-placed fibres makes them coupled. Subsequently, two degrees of freedom (bend radius and distance of the two fibres) enables wide tuneability of the coupled resonance properties

As with any proof-of-concept demonstration, it will be interesting to see which direction(s) this coupled special type of bottle resonator configuration will take. The authors make some suggestions in this regard, perhaps most exciting is replacing pure silica fibres with a material with high non-linearity or to use fibre twist as the third degree of freedom to achieve even more complex coupled-resonance systems, enabling to realize complex “photonics molecules”^7^. The authors also suggest to introduce a gap between the exciting fibre and the microresonators to boost the already-high Q by another two orders of magnitude. Such ultra-high Q in excess of 100 million are not necessary for a range of applications such as delay lines^8^, signal processors^9^, and microlasers^3,4^, but may open door to new applications such as tuneable repetition rate OFCs, cavity quantum electrodynamic (QED)^10,11^, and optomechanical applications^1,2^. In the future, it will be also interesting to see whether and how this research could be adopted to platforms that are more akin to integration and mass production such as photonics integrated circuits.
